# Industrial Co-Agglomeration and Air Pollution Reduction: An Empirical Evidence Based on Provincial Panel Data

**DOI:** 10.3390/ijerph182212097

**Published:** 2021-11-18

**Authors:** Rulong Zhuang, Kena Mi, Zhangwei Feng

**Affiliations:** 1 School of Business, Ningbo University, Ningbo 315211, China; zhuangrulong@nbu.edu.cn; 2 School of International Trade & Economics, Ningbo University of Finance & Economics, Ningbo 315175, China

**Keywords:** manufacturing, producer services, co-agglomeration, air pollution reduction, environmental impact

## Abstract

Industrial co-agglomeration plays a significant role in the moving up of the manufacturing industry in the value chain and in transforming China from a manufacturing giant into a world manufacturing power. This study establishes a co-aggregation index to explore spatio-temporal changes of the co-agglomeration between manufacturing and producer services in 30 provinces of China from 2004 to 2019. Furthermore, we use spatial Durbin model to analyze the impact of industrial co-agglomeration on air pollution reduction. We find that (1) the co-agglomeration index varies remarkably at spatio-temporal scale; (2) high co-agglomeration index is mainly distributed in eastern and central China, while low co-agglomeration index is mainly located in the western region; (3) the co-agglomeration index presents a cluster pattern among provinces, with the cluster of high value in eastern China and the cluster of low value in western China; and (4) the co-agglomeration between manufacturing and producer services is proven effetely to reduce air pollution, which is accompanied with spatial spillover effect. We also provided policy implications in line with diverse industries, multi hierarchies, and different regions, promoting the coordination of manufacturing and producer services and improving air quality.

## 1. Introduction

Since the twenty-first century, industrial co-agglomeration has increasingly become a worldwide trend, which is motivated by globalization and the new round of technological revolution and industrial transformation [1]. At present, although China has entered the post-industrialization stage, it is still a manufacturer of quantity rather than one of quality. A large number of problems need to be solved urgently, including product homogeneity, excess production capacity, weak innovation ability, low profitability, and low statue in global industrial value chain. One of the important reasons is the deagglomeration between manufacturing and producer services [2,3]. Meanwhile, China’s service industry, especially the producer services, is lagging behind and needs urgent upgrading. Under such circumstance, it is necessary to promote the coordinated development between manufacturing and producer services, which is helpful to build China as a manufacturer of quality.

Recently, since the environmental issues are more severe in China, the impact of co-agglomeration between manufacturing and producer services on the air quality has attracted widespread attention [4]. We systematically sorted out related literatures and divided these results into five aspects. The first aspect is to explore the theory of industrial co-agglomeration from the perspective of industrial agglomeration and its spatial externality [5,6]. The second aspect is the analysis of the formation process of industrial co-agglomeration using “three elements” from Marshall’s spatial economy [7,8]. The third aspect is to investigate the interaction between industrial co-agglomeration and spatial structure [9]. The fourth aspect is to calculate the industry co-agglomeration index through multiple methods. Finally, the last aspect is to explore the economic, social, and ecological impact of industrial co-agglomeration [10,11,12].

Although some research literatures have been produced, such as the impact of manufacturing agglomeration on air pollution, the impact of industrial co-agglomeration between manufacturing and producer services on the air quality is rarely explored [4,13]. Therefore, we raise following research questions:

(1) What are the spatio-temporal characteristics of the co-agglomeration between manufacturing and producer services in China?
(2) How does the industrial co-agglomeration affect air pollution?
(3) Are there spatial spillover effects and regional differences in the impact of co-agglomeration on air pollution?

**Figure 1:**
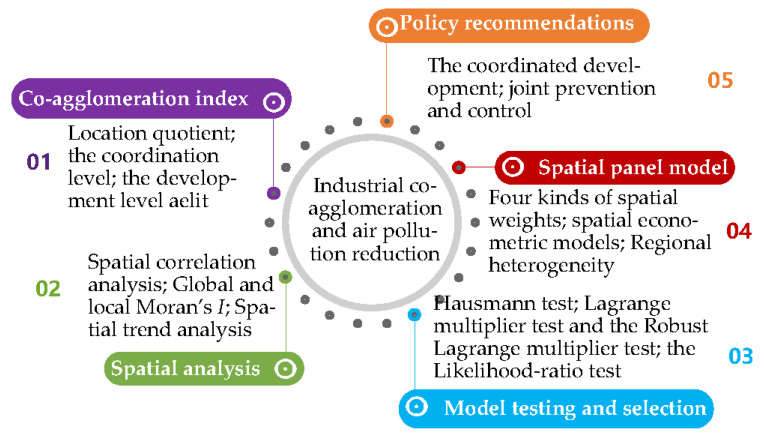
The research framework.

To answer these questions, we used 30 provinces in China as samples to discuss the spatio-temporal characteristics of the co-agglomeration between manufacturing and producer services and analyze its impact on air pollution using spatial econometric models based on the panel data from 2004–2019. Further, we also provided policy implications for the prevention and control of air pollution. The uniqueness and contributions of this study are as follows: first, we establish the co-agglomeration index between manufacturing and producer services to measure the degree of co-agglomeration of the two industries. Second, multiple pollutants are included to reflect pollution level. Third, we use spatial econometric models to examine the impact of co-agglomeration between manufacturing and producer services on air pollution using multiple spatial weighting matrices. Fourth, we analyze the spillover effects of the co-agglomeration. Furthermore, we obtain robust estimation results by using multiple econometric models and controlling the individual and time effects. Figure 1 illustrates the research framework.

## 2. Theoretical Analysis and Research Hypothesis

Producer services mainly provide production services for the manufacturing industry and plays a role of “service provider”, while the manufacturing industry is the “customers”. These industries often form complex and diversified horizontal or vertical economic links. In order to better serve “customers”, save transaction costs, and improve service quality, producer services often choose the location along with manufacturing; this spatial dependency produces the phenomenon of “co-agglomeration”.

Based on Marshall’s positive externality theory of industrial agglomeration, the impact mechanism of co-agglomeration between two industries on air pollution can be summarized into the following three aspects [14,15]. The first is the “service” effect. Producer services take human and knowledge capital as the main inputs and provide professional services with high added value, high technical content, low energy consumption, and low pollution for the manufacturing industry. As the producer services have run through all the value chain links of the manufacturing, the whole industrial chain can improve the production efficiency of the manufacturing industry, reduce the consumption of all kinds of resources, and promote the realization of green transformation so as to effectively reduce pollution emissions [16,17]. The second is the “forced force” effect. Producer services include research, design, information, finance, consulting, business, and other technical services, which can promote production specialization to be large and to expand capital and knowledge-intensive production so as to improve the productivity of labor and other factors of production. In this case, manufacturing enterprises are bound to be forced to carry out independent innovation and constantly improve their technological innovation capacity and professional production level. Simultaneously, they will pay more attention to ecological and environmental protection and green transformation, which is conductive to environmental pollution control. The third is the “learning” effect. Producer services are knowledge-, capital-, and technology-intensive industries, and their synergy with the manufacturing industry can increase face-to-face communication between enterprises or personnel and use formal or informal channels to exchange professional technologies, advanced knowledge, and ideas [18,19]. This process of knowledge spillover is also a process of continuous learning and progress, in which process can constantly improve innovation ability and technological level to promote transformation and upgrading and green development.

**Hypothesis** **1.** *Based on the panel data of provincial units, the co-agglomeration of manufacturing and producer services may also significantly reduce air pollution, but the regression symbol and direction are uncertain.*

At present, with the continuous improvement of the market economy, production factors can realize free flow and value generation between regions. The co-agglomeration of manufacturing and producer services will inevitably have spatial correlation and spillover effects [20]. Elements, such as knowledge, technology, and information, are concentrated in space, and there are more and more opportunities to contact and communicate with each other. In this case, different companies and different regions will form a stable foundation for communication and cooperation and gradually build a platform for technological exchange and cooperation [21]. The knowledge spillover effect generated by such external economies of scale can break through the limitations of space and improve the development level of manufacturing and producer services in the surrounding areas, thereby helping to alleviate regional environmental pollution. In addition, the regions that are close to each other tend to have the same development level. Similar resource endowments and cultural environment are conducive to inter-regional technology, capital, labor, knowledge, information, and other elements to play a role of spillover between regions, reducing the cost of element spillover [22].

**Hypothesis** **2.** *The level of co-agglomeration between the manufacturing and producer services is spatially correlated and can produce a spatial spillover effect on the reduction of air pollution.*

Due to the vastness of the territory and the characteristics of stepped distribution, the development levels of various regions in China are obviously different, resulting in significant regional differences in economic development stages and industrial structure layout. For example, according to economic and geographical conditions, China can be divided into eastern, central, and western regions as well as southern and northern regions. At the same time, China can also be divided into southeast and northwest regions based on the Hu Huanyong line. Due to the significant economic differences, the air pollution reduction effect of the co-agglomeration of manufacturing and producer services may also be regionally heterogeneous [23].

Specifically, the eastern region is close to the ocean and has a good geographical location advantage. At the same time, it is also a highly densely populated area. The various elements required for industrial development are relatively complete, and the development level of various industries is relatively high. Moreover, due to China’s active implementation of the industrial development strategy of industrial specialization and market integration, manufacturing companies continue to gather in the eastern region. Since the manufacturing industry can be regarded as the “customer” of the producer services to a certain extent, the development of the manufacturing can produce multiple economic links with the producer services, creating conditions for the development of the producer services. Therefore, the producer services gather in large numbers in the eastern region. In this context, the level of co-agglomeration of manufacturing and producer services in the eastern region may inevitably be higher than that in the central and western regions. The improvement of the co-agglomeration level is conducive to exerting agglomeration effects, enhancing corporate innovation capabilities and promoting green development, and increasing capital investment and technological innovation for air pollution reduction [24].

**Hypothesis** **3.** *Compared with the central and western regions, the pollution-reduction effect of the co-aggregation of manufacturing and producer services in the eastern region will be more significant. For the southern and northern regions and the southeast and northwest regions of the Hu Huanyong line, further investigation and verification are needed.*

## 3. Methods and Data Sources

### 3.1. Co-Agglomeration Index

We established the co-agglomeration index based on the agglomeration index of manufacturing and producer services, which are calculated by location quotient index, respectively. Location quotient index is widely used to show the degree of industry concentration and specialization. According to related research results [25,26], the co-aggregation index of manufacturing and producer services is:

(1) LQij=qijqjqiq

$LQ_{ij}$ is the agglomeration index of i industry in j province, where i is manufacturing (*man*) and producer services (*ser*), respectively, and j represents 30 provincial units. $q_{ij}$ represents the number of employments in the i industry in j province; $q_{j}$ is the employment number in j province; $q_{i}$ is the national i industry employment numbers; and q is the national employment numbers. On the basis of the above formula, the co-agglomeration index (xtjj) of manufacturing and producer services is further constructed, and the formula is as follows:

(2) $$xtjj=\left(1-\frac{\left|LQ_{man}-LQ_{ser}\right|}{LQ_{man}+LQ_{ser}}\right)+\left|LQ_{man}+LQ_{ser}\right|$$

where xtjj is the co-agglomeration index of manufacturing and producer services. It is obtained from the location quotient calculation of Formula (1). The co-agglomeration index consists of two dimensions. The first item on the right side of the equation represents the coordination level, and the second item represents the development level. The sum of the two items is the overall level of co-agglomeration. The higher the co-agglomeration index, the higher the level of coordination and development between manufacturing and producer services.

Due to incomplete industrial categories and inconsistent indicators in China, the co-agglomeration index of manufacturing and producer services can only reflect the relative level. If we replace the original data, the calculation results of the method may be slightly different. With the continuous development of manufacturing and producer services, the data of segmented industries will be a good choice to calculate so as to get a more realistic result from a micro perspective [13].

### 3.2. Spatial Correlation Analysis

We use Global Moran’s *I* to present the spatial correlations at global scale using the formula as follows [27]. The Global Moran’s *I* ranges from (−1,1). The index below 0 indicates negative correlation, that above 0 indicates positive correlation, and 0 indicates uncorrelation. Standardized statistics *Z* is commonly used to test for significant spatial autocorrelations in regions.

(3) $$I=\frac{n\sum_{i=1}^{n}\sum_{j=1}^{n}wij\left(xi-\bar{x}\right)\left(xj-\bar{x}\right)}{\sum_{i=1}^{n}\sum_{j=1}^{n}wij{\left(xi-\bar{x}\right)}^{2}}=\frac{\sum_{i=1}^{n}\sum_{j\neq i}^{n}wij\left(xi-\bar{x}\right)\left(xj-\bar{x}\right)}{S^{2}\sum_{i=1}^{n}\sum_{j\neq i}^{n}wij}$$

(4) $$S^{2}=\frac{1}{n}\sum_{i=1}^{n}{\left(xi-\bar{x}\right)}^{2}$$

where *I* is the Global Moran’s *I*, xi is the observation value of the region i, wij is the spatial weight matrix, and $S^{2}$ is the variance. When the Global Moran’s *I* is significantly positive, the index indicates positive spatial correlation; otherwise, the index indicates negative spatial correlation. When the Global Moran’s *I* is 0, it represents random spatial distribution.

Further, considering the existence of local spatial correlation, we use the Local Moran’s *I* (LISA) to reveal the local spatial characteristics of the co-agglomeration index using the formula as follows [28,29]. In the formula, it is the Local Moran’s *I* of each provincial unit, and the other variables have the same meaning as the Global Moran’s *I*.

(5) $$Ii=\frac{\left(xi-\bar{x}\right)}{S^{2}}\sum_{j}wij(xj-\bar{x})$$

### 3.3. Spatial Trend Analysis

We used trend analysis to visualize geographic features with large spatial span and transform the sampling point data of two-dimensional space into three-dimensional smooth curves, thus simulating the distribution laws and changing trends of geographic data in multiple directions. $Z_{i}(x_{i},y_{i})$ is the co-agglomeration index of $i.(x_{i},y_{i})$ is the planar right-angle coordinate system. The trend value is calculated from the following formula [30]. The formula is:

(6) $$Z_{i}(x_{i},y_{i})=T_{i}(x_{i},y_{i})+\varepsilon_{i}$$

(7) $$T_{i}(x_{i},y_{i})=\beta_{0}+\beta_{1}x+\beta_{2}y+\beta_{3}x^{2}+\beta_{4}y^{2}+\beta_{5}xy$$

where $T_{i}(x_{i},y_{i})$ is the spatial trend function used to calculate the fit trend values, and the study uses the second-order polynomial measure co-agglomeration index. Furthermore, $\varepsilon_{i}$ represents the stochastic error, reflecting the deviation between the true value of the co-agglomeration index and the trend value.

### 3.4. Construction of the Spatial Econometric Model

#### 3.4.1. Model Specification

IPAT model is applied to study the environmental impact of human activity, which is improved by Dietz and Rosa on STIRPAT stochastic model [31]. Based on STIRPAT stochastic model, this study conducted variable replacement to meet the research needs. Furthermore, research units may have spatial correlation and failed to satisfy the hypothesis of independent samples, the traditional methods may be not applicable [32]. Therefore, we used spatial econometric models to reflect spatial relations that can provide more realistic causal relations [33].

After careful consideration, there may be a strong spatial correlation between the co-agglomeration and air pollution. This situation cannot be ignored because it can affect the results of this study. Taking into account the diversity of spatial correlation, this paper uses the spatial Durbin model (SDM) to solve this problem. We estimated a SDM rather than a SAR or SEM. The advantage of SDM is that its spillover effects are flexible. It includes spatially lagged dependent variable and spatially lagged explanatory variables. The spatial correlation of different sources can be well reflected by SDM [34,35]. For the rigor of academic research, this article adds spatial dependency test and model selection test to further illustrate the applicability of the spatial Durbin model. In addition, we controlled for neighborhood and time-fixed effects. If these controls are not included, the spatial interaction effects and therefore the spillover effects may be biased, in most cases overestimated. Therefore, this paper mainly adopts SDM to carry out empirical test.

(8) $$\ln so_{2}{}_{it}=\rho W\ln so_{2}{}_{it}+\gamma \ln xtjj_{it}+\xi W\ln xtjj_{it}+\phi {X^{\prime }}_{it}+\zeta W{X^{\prime }}_{it}+a_{i}+\lambda_{t}+u_{it}$$

where $so_{2}$ is the sulfur dioxide emissions, xtjj means the industrial co-agglomeration index, and both variables are treated logarithmically. W is the weight matrix used to reflect the multiple spatial relations of provincial units where four classes are set: contiguity weights (IW), reverse distance weight (IW1), economic distance weight (IW2), and minimum neighbors weight (IW3). The setup method is no longer repeated here. ${X^{\prime }}_{it}$ is the control variable matrix, ϕ is the coefficient vector of control variables, and the lower angular scales *i* and *t* represent the provincial units and years, respectively. Furthermore, $a_{i}$ and $\lambda_{t}$ are individual and time fixing effects, respectively, and $u_{it}$ is the random perturbation. $W\ln so_{2}{}_{it}$, $W\ln xtjj_{it}$, and $W{X^{\prime }}_{it}$ are spatial lag terms of control variables, respectively, and ρ, ξ, and ζ are coefficient vectors.

In the regional heterogeneity analysis, we added the interaction of geographical location variables and industrial co-agglomeration. Model specification is defined as follows:

(9) $$\ln so_{2}{}_{it}=\rho W\ln so_{2}{}_{it}+\beta geo_{i}\times \ln xtjj_{it}+\xi Wgeo_{i}\times \ln xtjj_{it}+\phi {X^{\prime }}_{it}+\zeta W{X^{\prime }}_{it}+a_{i}+\lambda_{t}+u_{it}$$

In the above formula, $geo_{i}$ represents the geographical location, β represents the coefficients of the interaction term, and the remaining variables are consistent with the first model. The virtual variable of *east*, which represents the unit of the eastern region, is 1, and the remaining units are 0. The virtual variable of *ns*, which sets the unit belonging to northern region as 1, and the remaining units are 0. In terms of the Hu Huanyong line, the virtual variable is set as *hhy*. When the research unit is located on the southeast side, the variable is set to 1. When the research unit is located on the northwest side, the variable is set to 0.

#### 3.4.2. Variable Descriptions

(1) Dependent variable: average annual concentration of sulfur dioxide (ug/m^3^). Industrial pollution refers to the “three wastes” (waste water, waste gas, waste residue) and various noise. Sulfur dioxide is a typical pollutant of air pollution. China’s energy consumption structure is based on coal and oil that produce sulfur dioxide and other harmful gases [36]. Due to the lack of a widely recognized indicator, SO_2_ was selected as a representative indicator of air pollution [37].

(2) Independent variable: industrial co-agglomeration index (xtjj) [4,13,38]. The industrial agglomeration index includes manufacturing and producer services. Producer services exist for promoting technological progress and industrial upgrading and improve production efficiency for the manufacturing industry. It is an emerging industry that originates and develops independently from the manufacturing industry, which does not directly provide services to consumers. Due to the rapid development, scholars have yet to reach an agreement on the definition of producer services [39]. According to existing literatures, producer services can be classified into five categories: transportation, warehousing and postal services, information transmission, computer service and software, finance, leasing and business services, and scientific research and technology services.

(3) Control variables: there are various factors affecting air quality. The following variables were also selected as the control variables: (i) Foreign investment (*wstzbz*). According to the theory of “pollution paradise”, foreign investment is also one of the important factors affecting the air pollution in the destination country. The proportion of foreign investment in GDP was used. (ii) Industrial structure (*ecbz*). Whether industrial structure is advanced is an important factor affecting air pollution. Advanced industrial structure not only promotes sustainable economic and social development but also reduces air pollution. The proportion of secondary industry output value in GDP was used. (iii) Traffic conditions (*glmd*). Transportation consumes a large amount of energy and discharges air pollutants, which is also an important factor affecting air quality. The highway density was used. (iv) Technology innovation (*shouq*). Advanced technology can improve production efficiency, improve pollution control level, and reduce pollutant emissions. The amount of patent application authorization was used. (v) Environmental regulation (*zlfy*). Many studies have shown that environmental regulations help to improve air quality. Therefore, we used industrial waste gas treatment and operation costs.

### 3.5. Data Sources

Due to a lack of data from Tibet, Hong Kong, Macao, and Taiwan, this study used 30 provincial units in China. We obtained the air pollution data from China Statistical Yearbook (all Statistical Yearbooks can be accessed on http://www.stats.gov.cn/tjsj/ndsj/, accessed on 20 August 2021), China Environmental Statistical Yearbook, and China air quality online monitoring and analysis platform (accessed on https://www.aqistudy.cn/, accessed on 20 August 2021). We derived the data related to industrial co-agglomeration from China Statistics Yearbook, China Labor Statistics Yearbook, and China Population and Employment Statistics Yearbook, etc. We used the data of 2004–2019 to ensure data consistency and uniform statistical standard.

According to the research period and samples, the data structure of this article is a balanced panel data. By obtaining data from a variety of official statistical yearbooks and databases, this article can ensure data integrity, consistency, continuity, and credibility. In order to eliminate possible problems, such as heteroscedasticity and variable unit inconsistency, the logarithm of variable was processed. Furthermore, this paper eliminates the research samples with missing data and reasonably eliminates outliers so that the data can better fit the model.

## 4. Temporal and Spatial Characteristics

### 4.1. General Characteristic Analysis

We calculated the agglomeration index of 30 provinces from 2004–2019, and thus, we obtained the coordination level and development level, respectively. To reflect the characteristics of each province, the mean values were calculated and are shown in Table 1. In general, there is a variation between the manufacturing agglomeration index and the producer services agglomeration index in different provinces, which brings about significant interprovincial differences in the co-agglomeration index.

Eastern provinces, including Guangdong, Fujian, Jiangsu, Tianjin, Zhejiang, and Shandong, have high manufacturing agglomeration index. By contrast, Hainan, Xinjiang, Beijing, Heilongjiang, Inner Mongolia, and Guizhou are lower. Hainan is the last and is about a fifth of Guangdong. In terms of agglomeration index of producer services, Beijing is the highest and has an absolute advantage in the country, followed by Shanghai and Tianjin, which also have obvious advantages. In contrast, Fujian, Shandong, Henan, Guizhou, Jiangxi, etc. are lower. Beijing is about four times the number of bottom-ranked Fujian. It reveals that there are significant interprovincial differences, which further shows the imbalance in the development of these two industries in the whole country.

The inter-provincial differences of coordination and development level are also evident. From the perspective of coordination level, Hubei’s level is highest, followed by Anhui, Liaoning, Hebei, Sichuan, Hunan, etc. Due to the similar agglomeration level of two industries in the country, these provinces can obtain a higher coordination level. However, the coordination level of Beijing, Hainan, Xinjiang, Fujian, and Qinghai is low, which shows that the agglomeration level of manufacturing and producer services in these provinces is greatly different in the country. Take Beijing as an example; Beijing’s manufacturing agglomeration index is only 0.53, while the agglomeration index of producer services is 2.55. Therefore, Beijing’s manufacturing industry does not have an advantage in the whole country, while the producer services have an absolute advantage, which also indirectly reflects Beijing’s advanced industrial structure. From the perspective of development level, Beijing, Shanghai, Guangdong, Tianjin, Jiangsu, Fujian, and Zhejiang are all in the forefront, indicating that these provincial units have obvious advantages in the field of manufacturing or producer services in the country so as to reach a high level of development level.

Finally, the top 10 co-agglomeration indices are for Shanghai, Beijing, Tianjin, Guangdong, Liaoning, Jiangsu, Zhejiang, Fujian, Jilin, and Hubei. The co-agglomeration indices are all above 2.75. Among them, the co-agglomeration indices of top five provinces are all more than 3, indicating that the two industries have a high level of collaborative development and showing a trend of mutual promotion and mutual improvement. In contrast, Hainan, Xinjiang, Guizhou, Heilongjiang, Inner Mongolia, Yunnan, Gansu, Shanxi, Ningxia, and Qinghai belong to the last 10. The co-agglomeration indices are all below 2.5, indicating that the two industries are relatively low and need to be further improved.

**Figure 2:**
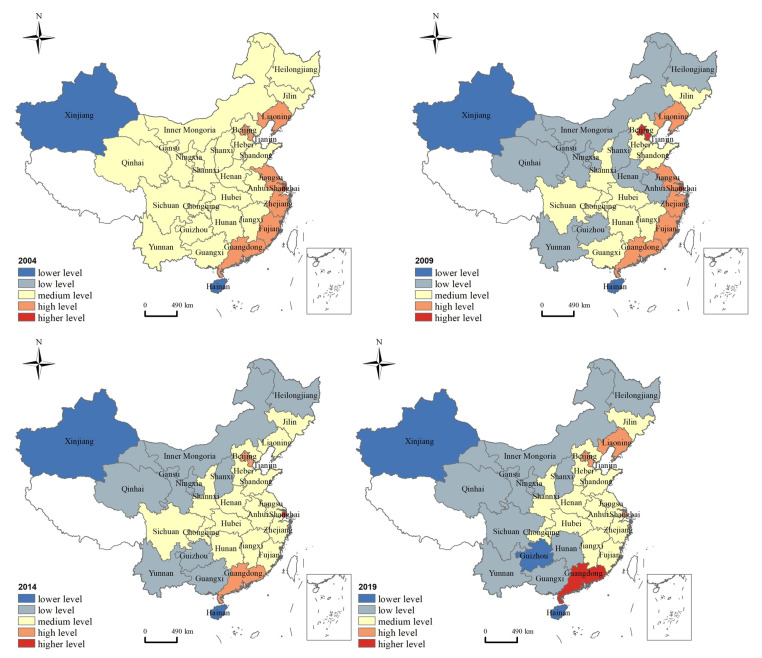
Spatio-temporal differences of co-agglomeration index between the manufacturing and producer services.

We selected the co-agglomeration index of 30 provinces for spatial visualization in typical yearsd and divide them into five types according to the natural breakpoint classification. They are lower level (1.5, 2), low level (2, 2.5), medium level (2.5, 3), high level (3, 3.5), and higher level (3.5, 4). Figure 2 reveals the spatio-temporal variation of the co-agglomeration index. Firstly, the provinces with high or higher level of co-agglomeration are mainly distributed in the eastern and central regions, while the lower or low level of co-agglomeration are mainly located in the western region. Secondly, the three types of provincial units with high level, medium level, and low level changed greatly.

The reasons for this phenomenon are explained as follows. In the eastern region, due to location advantages, economic foundation, and policy support, the manufacturing has a relatively high level. Therefore, it continues to generate a variety of service demands, which promotes the rapid development of various producer services, for example, the financial industry, information technology industry, consulting industry, etc. In this process, the manufacturing and producer services have continuously strengthened cooperation and exchanges, and the level of co-agglomeration has been continuously improved. At present, the co-agglomeration level in the eastern regions is in a leading position in the country. In the central region, due to the radiation and driving effect of the eastern region, the spillover effect of the industry continues to appear, which gradually has led to the development of co-agglomeration level. In contrast, due to the lack of a strong industrial foundation, market, and location conditions, the manufacturing and producer services of the western region are relatively backward. Although the local government is also trying to improve it, they have not formed an interactive trend. Therefore, its co-agglomeration level is low.

Take Shanghai as an example; with good location conditions, strong economic foundation, and policy support, Shanghai’s advanced manufacturing and producer services have been developing rapidly. With the continuous emergence of various manufacturing bases, the producer services has also begun to form an agglomeration area dominated by key enterprises. They focus on R&D and design, business services, marketing, after-sales service, and other services. They take the manufacturing as the main service object and provide personalized and high-quality service products. It is helpful to the efficiency improvement, transformation, and upgrading of the manufacturing. In this process, manufacturing and producer services can achieve mutual promotion and co-agglomeration development.

In 2004, the co-agglomeration index for 30 provinces consisted of four types: higher, high, medium, and low. Shanghai is on higher level, while provinces with higher level are mainly distributed in the eastern coastal areas, including Liaoning, Beijing, Tianjin, Jiangsu, Zhejiang, Fujian, etc. Most provinces in the central and western regions belong to the middle level. In addition, Xinjiang and Hainan are at lower levels. In 2009, Beijing and Tianjin belonged to higher level. Xinjiang and Hainan are still at the lower level of co-agglomeration. In 2014, changes were not remarkable. Beijing and Tianjin decreased from higher to high level, and Liaoning, Jiangsu, Zhejiang, and Fujian also dropped from high to medium level. In contrast, Anhui and Henan rose from low to medium level. In 2019, Guangdong replaced Shanghai as the only province with a higher level of co-agglomeration. Sichuan and Guizhou showed a trend of hierarchical decline, with Sichuan falling from medium to a low level, while Guizhou fell to a lower level. Liaoning rose from a medium to high level.

### 4.2. Spatial Correlation Analysis

Table 2 shows the results of spatial correlation of industrial co-agglomeration index during 2004–2019. The Global Moran’s *I* is positive, and all have passed the significance tests, indicating a positive spatial correlation for industrial co-agglomeration index. In 2004, the *p*-value was 0.0512. From 2005 to 2014, the *p*-value was 0.01–0.05. From 2015 to 2019, the *p*-value was below 0.01. The above data show that the level of significance continues to increase over time. Meanwhile, the Global Moran’s *I* shows a rising fluctuation trend, indicating that the spatial correlation trend of the co-agglomeration index is continuously increasing.

**Figure 3:**
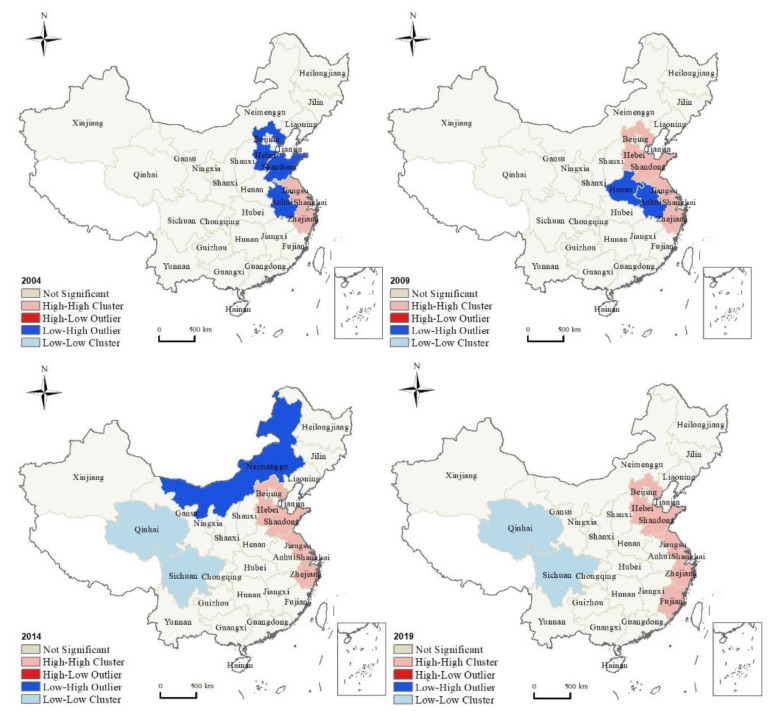
Local Moran’s *I* of the co-agglomeration index of 30 provinces.

The Local Moran’s *I* for the co-agglomeration index of the 30 provinces is further analyzed. Results are shown in Figure 3. In general, there are significant high and low-value cluster areas. The high-value cluster area is constantly expanding, and the spatial characteristics of polarization are formed in the eastern and western regions.

In 2004, there were only two types of “high-high” cluster and “low-high” cluster. There are three provinces of “high-high” clusters—Jiangsu, Shanghai, and Zhejiang—while Anhui, Shandong, and Hebei belong to the “low-high” type. In 2009, the provinces of “high-high” increased, and Shandong and Hebei joined them. The “low-high” types were only Anhui and Henan. In 2014, the inter-provincial differences began to exhibit polarization characteristics, and the cluster types changed from two to three. The provinces of “high-high” type were further increased, and Anhui joined it. It changed from three in 2004 to six in 2014. At the same time, Inner Mongolia, adjacent to northern Hebei, became a “low-high”. It is worth noting that Qinghai and Sichuan belong to the “low-low” type. The low-value cluster area in the southwest and the high-value cluster area in the east form the polarized spatial characteristics. In 2019, provincial units of “high-high” were further increased, and Fujian joined it. At the same time, low-value clusters with Qinghai and Sichuan still exist.

### 4.3. Spatial Trend Analysis

**Figure 4:**
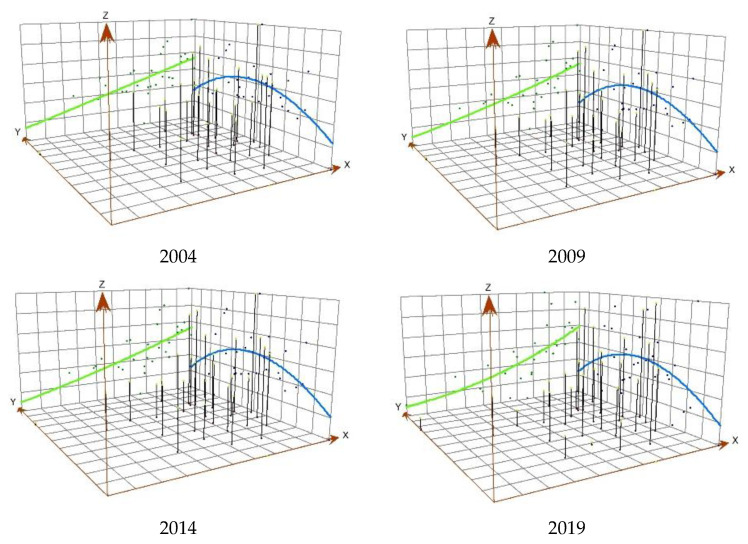
Trend analysis of the co-agglomeration index of 30 provinces.

To explore the spatial variation of the co-agglomeration index, a trend analysis was conducted by using ArcGIS10.6. Results are shown in Figure 4. In general, the co-agglomeration index of 30 provinces presents the spatial characteristics of “high in the northeast, central uplift, and low in the southwest”.

Specifically, the fitted trend line maintains the sloped spatial structure characteristic of “high in east and low in west”, indicating that the co-agglomeration index of the east is higher than that of the central region, while the central region is higher than that of the west. Moreover, due to the rise in the co-agglomeration index in the eastern region, the oblique trend line began to change to the U-type direction. The reason for this change in trend is that the co-agglomeration index of Beijing, Tianjin, Shanghai, Zhejiang, and Jiangsu in the eastern region has been rising in recent years, thus leading to the overall spatial structure of the east-west direction, to a certain extent. In the north-south direction, the fitting trend line maintains the inverted U spatial structure characteristics of “low on both sides, high in the middle”, indicating that the co-agglomeration index of provincial units in the central region is higher than that in the north and south. The reason for this phenomenon is that co-agglomeration index in the northern and southern provinces is low, such as Inner Mongolia, Heilongjiang, Guangxi, Yunnan, Hainan, etc., while the central Beijing, Tianjin, Shanghai, Jiangsu, and Zhejiang are high-value index, thus forming the inverted U structure characteristics.

## 5. Empirical Results

### 5.1. Model Selections

Through the above analysis, the co-agglomeration index shows a significant spatial correlation relationships. Moreover, the spatial correlation of sulfur dioxide as a typical pollutant of air pollution has already been verified. Therefore, the spatial econometric model is an appropriate choice to analyze the air reduction effect of industrial co-agglomeration. The results are shown in Table 3. First, it was found by the Hausmann test that the results significantly reject the original hypothesis, suggesting that the fixed effect should be chosen. We choose the double fixed effect of time and individual. Further, the Lagrange Multiplier Test (LM) and the Robust Lagrange Multiplier Test (Robust LM) were used to judge the suitability of the spatial lag and spatial error models. Results show that both passed the 1% significance test, indicating that both can be adapted to model estimation. However, the larger statistics of the spatial lag model were more advantageous. Further, the Likelihood-Ratio Test (LR) found that the spatial Durbin model with more general properties cannot be reduced to a spatial lag or spatial error model; in other words, the spatial Durbin model should be chosen.

### 5.2. The Baseline Regression Results

Table 4 reports the regression results of the air pollution reduction effect of industrial co-agglomeration under different spatial weights. Given the apparent time dependence of air pollution, this study incorporates the first-phase lag values of the interpreted variable sulfur dioxide into the spatial Durbin model.

The results show that no matter the use of contiguity weight, reverse distance weight, economic distance weight, or the minimum neighbors weight, the co-agglomeration between two industries can significantly reduce the sulfur dioxide emission. In other words, the improvement of industrial co-agglomeration can effectively reduce air pollution. It is worth noting that the spatial lag term of co-agglomeration is also significantly negative under different spatial weight matrices, indicating that the pollution-emission reduction effect of industrial co-agglomeration has a significant spillover effect. Specifically, the estimated coefficients of industrial co-agglomeration fluctuate between 0.530 and 0.942 under different spatial weight matrices, and all passed the 1% significance test. Take the model (1) as an example; increasing 1% of the co-agglomeration index will decrease by 0.586% sulfur dioxide emission. Meanwhile, the coefficient of the sulfur dioxide first-phase lag term is significantly positive at the 1% level, which verifies the time dependence and further demonstrates the long-term and difficulty of air pollution control.

In addition, from the perspective of control variables, the variable of *wstzbz* can significantly reduce sulfur dioxide emissions, perhaps due to the advanced production technology, experience, and concepts brought about by foreign investment in the process of production and operation. The variables of *glmd* and *ecbz* have a positive impact on it. The increase of highway density is mostly due to the increase of vehicles, and excessive vehicles, especially private vehicles, are bound to increase air pollution by increasing energy consumption and exhaust emissions. Most of the secondary industry is manufacturing, in which is easy to produce polluted waste gas. Therefore, the increase of the proportion of the secondary industry will increase the burden of air pollution. From a national point of view, the optimization and upgrading of industrial structure still have a long way to go. In addition, the amount of patent application authorization represents the level of technological innovation. Technological innovation is an important channel for green development; therefore, it is an important force to achieve air pollution-emission reduction.

### 5.3. Analysis of Regional Heterogeneity

Due to the different local economic and social development foundations, the air pollution reduction effect of industrial co-agglomeration may remain regionally heterogeneous. Therefore, we attempted to test regional heterogeneity by three types. The results are shown in Table 5.

(1) Variation between eastern and mid-western. Column (1) in Table 5 shows that industrial co-agglomeration in the eastern regions has a more significant emission-reduction effect on sulfur dioxide than in the central and western regions. That is to say, improving industrial co-agglomeration in eastern regions will show an obvious effect. Due to rapid economic and social development in the eastern region, the agglomeration of the manufacturing industry is also far higher than that in the central and western regions, which also easily leads to the discharge of air pollutants, such as sulfur oxides, nitrogen oxides, and inhalable particulate matter. In this case, if the development of the producer services in the eastern region is accelerated, and special attention is paid to the coordination between two industries; this will help to reduce air pollution effectively.

(2) Variation between northern and southern region. The Qinling–Huaihe line is the boundary of the north and the south in the conventional sense, and the natural conditions, production mode, and local customs are completely different. What is particularly obvious is that there are natural and geographical differences between the north and the south, and the different natural conditions, such as temperature, humidity, and precipitation, have different effects on air pollution. Column (2) in Table 5 shows that industrial co-agglomeration in the south is more evident for sulfur dioxide emissions reduction than that in the north. The possible reason is that the manufacturing industry in the south is more industrialized compared with the north. This inevitably leads to an increase in air pollutants emissions, such as sulfur dioxide. In contrast, producer services are lagging behind. Therefore, improving and accelerating the coordinated development of the two industries will have a significant effect on air pollution reduction. On the other hand, natural weather conditions, such as humidity saturation, static weather, and radiation temperature inversion, in the south will also lead to aggravated air pollution [40,41]. Therefore, the improvement of industrial co-agglomeration will help to reduce sulfur dioxide emissions, thus making a more significant improvement in air quality.

(3) Variation along both sides of the Hu Huanyong line. The Hu Huanyong line is the population density dividing line proposed by the famous geographer Hu Huanyong, which is roughly inclined 45 degrees straight line. The Hu Huanyong line divides the land into the southeast and the northwest according to the population density. Column (3) in Table 5 shows that the air pollution reduction effect of industrial co-agglomeration on both sides does indeed show differences, and the improvement of industrial co-agglomeration level in the southeast side will have a more significant effect on air pollution reduction. The southeast side belongs to the region with an advanced economy and large population, and the manufacturing industry and producer services are relatively developed, which plays an obvious role in air pollution reduction. The economic and society of the northwest side of the Hu Huanyong line is relatively backward, and the promotion effect of producer services on the manufacturing industry is limited. Therefore, the role of air pollution reduction is not as effective as that of the southeast side of the Hu Huanyong line.

### 5.4. Robustness and Endogeneity Test

To evaluate the effectiveness of the above baseline regression results, it was tested for robustness by means of replacing variables and models, and the results are as shown in Table 6.

The air pollution is measured by the comprehensive index. Considering that industrial pollutants are an important source of air pollution in China, we used three air pollutants (sulfur dioxide, nitrogen dioxide, inhalable particulate matter) to construct index system and used entropy method to calculate the comprehensive index. Similarly, considering the time dependence of air pollution, we incorporated the first-phase lag of the comprehensive index into the regression. Columns (1)–(4) show that co-agglomeration index still has a significant negative effect under different spatial weight matrix. Compared with a single pollutant, the coefficient is between 0.017 and 0.024. Moreover, the first-phase lag term of the comprehensive index is significantly positive at the 1% level, further confirming the time dependence. Moreover, since the spatial lag model was found during the model selection process and shows good adaptability, the spatial lag model was further selected for the robustness test. Columns (5)–(8) show that spatial lag models can get basically consistent conclusions under different spatial weight matrices.

The endogenous problems may bring partial estimation errors. In order to alleviate the endogenous problems due to many reasons, we lagged the core explanatory variables and control variables for one period and then estimated. Meanwhile, considering that the fixed-effect model can alleviate the possible endogenous problems due to variable omission, we still used the spatial Durbin model and the spatial lag model under the dual fixed effect for model estimation. Table 7 shows the results of endogenous treatment. Both models still show that the co-agglomeration of two industries plays a significant role in reducing air pollution. It shows that significant causality remains true while considering endogenous problems.

## 6. Conclusions and Discussions

Through the above research, we obtained the following conclusions: (1) there are obvious differences between the manufacturing agglomeration index and the producer services agglomeration index in different provincial units, which brings about significant interprovincial differences in the co-agglomeration index. (2) Provincial units with higher or high levels of co-agglomeration index are distributed in the east and central regions, while provincial units with lower or low levels of co-agglomeration index are mainly located in the western regions. (3) Co-agglomeration index has spatially positive correlation between inter-provinces, and provincial units with high (or low) co-agglomeration index present spatial agglomeration characteristics. (4) Locally, there are significantly high and low value cluster areas, and the high-value cluster area is constantly expanding, and the spatial characteristics of polarization of the co-agglomeration index are formed in the eastern and western regions. (5) From the perspective of the spatial variation trend, the co-agglomeration index of 30 provincial units presents the spatial characteristics of “high in the northeast, central uplift, and low in the southwest”. (6) In the east-west direction, the fitting trend line maintains the oblique space structure characteristics of “east high and west low”. In the north-south direction, the fitting trend line maintains the inverted U spatial structure characteristics of “low at both sides and high in the middle”. (7) No matter the use of contiguity weight, reverse distance weight, economic distance weight, or the minimum neighbors weight, the co-agglomeration index of manufacturing and producer services can significantly reduce the emission of sulfur dioxide. In other words, the improvement of industrial co-agglomeration level can effectively reduce the level of air pollution. (8) It is worth noting that the co-agglomeration spatial lag term is also significantly negative under different spatial weight matrix, which indicates that the pollution emission reduction effect creates a significant spillover effect.

This study expands and deepens the research on the air pollution reduction effect of the co-agglomeration between manufacturing and producer services and provides new empirical evidence and inspiration for air pollution control in China. We propose the following policy suggestions according to the research conclusions:

Firstly, from the perspective of the pollution reduction effect, central government should strengthen top-level design, deepen industrial reforms, and eliminate institutional obstacles that may be encountered in the process of industrial co-agglomeration. Specifically, local government should continuously improve industrial planning and guide industrial agglomeration in an environmentally friendly way. In addition, the government should encourage enterprises from different industries to continuously carry out technological innovations to reduce the emission of various pollutants so as to promote the realization of a sustainable society. For example, the central and local governments should continue to introduce various policies for companies to adopt environmentally friendly production methods.

Secondly, due to the significant differences between provinces, personalized measures should be taken to promote the co-agglomeration in manufacturing and producer services. The eastern provinces are competitive in advanced production factors, while western provinces relatively lack this quality. Therefore, it is necessary to integrate different industrial advantages from different regions to promote the industrial co-agglomeration. The eastern provinces should focus on advanced producer services and innovate the development model with the manufacturing. On the basis of their own conditions, the central and western provinces should strengthen the industrial relations and cooperation with the eastern provinces in different fields and levels to improve the level of co-agglomeration. During this process, efforts from different regions should be made to promote the upgrading of industrial structure and the transformation of production methods so as to contribute to the ecological environment protection.

Thirdly, the study found that air pollution has significant spatio-temporal dependence. Therefore, it is necessary to pay attention to the joint prevention and control. On the one hand, local governments from different regions need to enhance cooperation and establish environmental monitoring and control platforms, such as Air Pollution Joint Prevention and Control Platform in the Yangtze River Delta. On the other hand, the spatial spillover effect of co-agglomeration on pollution reduction cannot be ignored. From the perspective of environmental friendliness, each province should improve the level of co-agglomeration between manufacturing and producer services, which will not only help improve its own environmental quality but will also improve the environmental quality of other regions.

## Figures and Tables

**Table 1:** Analysis of the co-agglomeration index of manufacturing and producer services.

Province	Manufacturing Agglomeration Index	Producer Services Agglomeration Index	Coordination Level	Development Level	Co-Agglomeration Index
Beijing	0.53	2.55	0.35	3.08	3.43
Tianjin	1.32	1.19	0.90	2.51	3.41
Hebei	0.81	0.95	0.92	1.76	2.68
Shanxi	0.62	0.93	0.80	1.55	2.34
Inner Mongolia	0.56	1.05	0.69	1.61	2.30
Liaoning	0.97	1.12	0.93	2.10	3.02
Jilin	0.84	1.08	0.88	1.91	2.79
Heilongjiang	0.54	0.99	0.71	1.54	2.25
Shanghai	1.20	1.82	0.79	3.02	3.82
Jiangsu	1.45	0.81	0.72	2.26	2.97
Zhejiang	1.28	0.87	0.81	2.15	2.96
Anhui	0.79	0.85	0.93	1.64	2.57
Fujian	1.52	0.69	0.63	2.21	2.85
Jiangxi	0.94	0.80	0.89	1.74	2.63
Shandong	1.26	0.73	0.74	1.99	2.73
Henan	0.91	0.74	0.88	1.65	2.54
Hubei	0.95	0.89	0.94	1.83	2.77
Hunan	0.74	0.89	0.91	1.63	2.53
Guangdong	1.61	0.98	0.76	2.59	3.35
Guangxi	0.68	0.99	0.81	1.67	2.48
Hainan	0.33	1.00	0.50	1.33	1.83
Chongqing	0.82	1.03	0.88	1.85	2.73
Sichuan	0.77	0.91	0.92	1.68	2.59
Guizhou	0.57	0.78	0.84	1.36	2.20
Yunnan	0.62	0.84	0.85	1.46	2.31
Shanxi	0.80	1.08	0.85	1.88	2.73
Gansu	0.61	0.90	0.80	1.51	2.32
Qinghai	0.61	1.18	0.69	1.79	2.47
Ningxia	0.64	1.03	0.76	1.66	2.43
Xinjiang	0.38	0.85	0.62	1.24	1.86

**Table 2:** Global Moran’s *I* of the co-agglomeration index of 30 provinces.

Year	Global Moran’s *I*	Z	P	Year	Global Moran’s *I*	Z	P
2004	0.1115	1.9494	0.0512	2012	0.1380	2.2781	0.0227
2005	0.1279	2.1559	0.0311	2013	0.1384	2.2966	0.0216
2006	0.1475	2.4189	0.0156	2014	0.1583	2.5509	0.0107
2007	0.1596	2.5914	0.0096	2015	0.1770	2.7891	0.0053
2008	0.1538	2.5076	0.0122	2016	0.1929	2.9911	0.0028
2009	0.1499	2.4284	0.0152	2017	0.1749	2.7477	0.0060
2010	0.1478	2.4234	0.0154	2018	0.1645	2.6131	0.0090
2011	0.1220	2.0728	0.0382	2019	0.1955	3.0022	0.0027

**Table 3:** Model selection test results.

Methods	Statistics	*p*
Hausman	52.18	0.000
LM-Spatial error	14.787	0.000
Robust LM-Spatial error	82.993	0.000
LM-Spatial lag	120.703	0.000
Robust LM-Spatial lag	188.909	0.000
LR-Spatial error	66.39	0.000
LR-Spatial lag	64.81	0.000
LR-SDM-ind	51.53	0.000
LR-SDM-time	303.52	0.000

**Table 4:** The empirical results of the baseline model.

	(1)	(2)	(3)	(4)
	(SDM-IW)	(SDM-IW1)	(SDM-IW2)	(SDM-IW3)
L.lnSo2	0.891 ***	0.863 ***	0.879 ***	0.843 ***
	(0.027)	(0.026)	(0.025)	(0.026)
lnXtjj	−0.586 ***	−0.942 ***	−0.530 ***	−0.724 ***
	(0.199)	(0.212)	(0.192)	(0.192)
Wstzbz	−0.339 **	−0.450 ***	−0.355 **	−0.356 **
	(0.171)	(0.172)	(0.165)	(0.164)
lnGlmd	0.273 ***	0.187 **	0.220 ***	0.205 **
	(0.088)	(0.083)	(0.081)	(0.084)
Ecbz	0.138	0.048	−0.002	−0.060
	(0.253)	(0.253)	(0.251)	(0.254)
lnShouq	−0.056 *	−0.071 **	−0.051	−0.076 **
	(0.034)	(0.031)	(0.033)	(0.031)
lnZlfy	−0.019	−0.030	−0.009	−0.014
	(0.024)	(0.024)	(0.023)	(0.023)
W*Lnxtjj	−0.756 **	−7.625 ***	−1.142 ***	−1.429 ***
W*Y	0.067 *	0.089	0.101 ***	0.026
	(0.039)	(0.128)	(0.032)	(0.041)
W*X’	Yes	Yes	Yes	Yes
Ind fixed	Yes	Yes	Yes	Yes
Time fixed	Yes	Yes	Yes	Yes
N	450	450	450	450
r2_a	0.8824	0.6352	0.8192	0.7586

Note: Robust standard errors in parentheses. * *p* < 0.1, ** *p* < 0.05, *** *p* < 0.01; the following tables are the same.

**Table 5:** The empirical results of regional heterogeneity.

	(1)	(2)	(3)
	ln*so2*	ln*so2*	ln*so2*
lnXtjj_east	1.247 **		
	(0.572)		
lnXtjj_ns		−0.833 *	
		(0.446)	
lnXtjj_hhy			0.783 **
			(0.374)
W*Y	0.270 ***	0.259 ***	0.251 ***
	(0.052)	(0.054)	(0.053)
X’	Yes	Yes	Yes
W*X’	Yes	Yes	Yes
Ind fixed	Yes	Yes	Yes
Time fixed	Yes	Yes	Yes
N	480	480	480
r2_a	0.5102	0.4448	0.5948

* *p* < 0.1, ** *p* < 0.05, *** *p* < 0.01.

**Table 6:** The empirical results of robust test.

	(SDM- IW)	(SDM- IW1)	(SDM- IW2)	(SDM- IW3)	(SAR- IW)	(SAR- IW1)	(SAR- IW2)	(SAR- IW3)
	(1)	(2)	(3)	(4)	(5)	(6)	(7)	(8)
lnXtjj	−0.017 *	−0.024 **	−0.019 **	−0.023 ***	−0.510 ***	−0.500 ***	−0.491 ***	−0.490 **
	(0.009)	(0.010)	(0.009)	(0.009)	(0.190)	(0.191)	(0.190)	(0.191)
L.lnZhz	0.892 ***	0.873 ***	0.902 ***	0.868 ***				
	(0.029)	(0.028)	(0.028)	(0.029)				
L.lnSo2					0.871 ***	0.882 ***	0.872 ***	0.875 ***
					(0.025)	(0.024)	(0.025)	(0.025)
X’	Yes	Yes	Yes	Yes	Yes	Yes	Yes	Yes
W*X’	Yes	Yes	Yes	Yes	Yes	Yes	Yes	Yes
W*Y	Yes	Yes	Yes	Yes	Yes	Yes	Yes	Yes
Ind fixed	Yes	Yes	Yes	Yes	Yes	Yes	Yes	Yes
Time fixed	Yes	Yes	Yes	Yes	Yes	Yes	Yes	Yes
N	450	450	450	450	450	450	450	450
r2_a	0.6847	0.4878	0.6376	0.5013	0.8738	0.8817	0.8752	0.8787

* *p* < 0.1, ** *p* < 0.05, *** *p* < 0.01.

**Table 7:** The results of endogenous treatment.

	(SDM- IW)	(SDM- IW1)	(SDM- IW2)	(SDM- IW3)	(SAR- IW)	(SAR- IW1)	(SAR- IW2)	(SAR- IW3)
	(1)	(2)	(3)	(4)	(5)	(6)	(7)	(8)
lnXtjj_1	−0.693 ***	−1.102 ***	−0.615 ***	−0.793 ***	−0.565 ***	−0.559 ***	−0.543 ***	−0.541 ***
	(0.215)	(0.230)	(0.207)	(0.209)	(0.206)	(0.207)	(0.207)	(0.207)
X’	Yes	Yes	Yes	Yes	Yes	Yes	Yes	Yes
W*X’	Yes	Yes	Yes	Yes	Yes	Yes	Yes	Yes
W*Y	Yes	Yes	Yes	Yes	Yes	Yes	Yes	Yes
Ind fixed	Yes	Yes	Yes	Yes	Yes	Yes	Yes	Yes
Time fixed	Yes	Yes	Yes	Yes	Yes	Yes	Yes	Yes
N	420	420	420	420	420	420	420	420
r2_a	0.8418	0.5409	0.7821	0.7301	0.8643	0.8739	0.8646	0.8696

*** *p* < 0.01.

## Data Availability

No new data were created or analyzed in this study. Data sharing is not applicable to this article.
